# Structured CT reporting improves accuracy in diagnosing internal herniation after laparoscopic Roux-en-Y gastric bypass

**DOI:** 10.1007/s00330-020-06688-x

**Published:** 2020-02-20

**Authors:** Jeannette C. Ederveen, Simon W. Nienhuijs, Saskia Jol, Simon G.F. Robben, Joost Nederend

**Affiliations:** 1 Department of Radiology, Maastricht University Medical Centre, PO Box 5800, 6202 AZ Maastricht, The Netherlands; 2 Department of Surgery, Catharina Hospital Eindhoven, Michelangelolaan 2, 5623 EJ Eindhoven, The Netherlands; 3 Department of Radiology, Amsterdam University Medical Centres location VUmc, Amsterdam, The Netherlands; 4 Department of Radiology, Catharina Hospital Eindhoven, Michelangelolaan 2, 5623 EJ Eindhoven, The Netherlands

**Keywords:** Hernia, Gastric bypass, Tomography, X-ray computed, Sensitivity and specificity

## Abstract

**Objectives:**

To confirm that structured reporting of CT scans using ten signs in clinical practice leads to a better accuracy in diagnosing internal herniation (IH) after gastric bypass surgery, compared with free-text reporting.

**Methods:**

In this prospective study, CT scans between June 1, 2017, and December 1, 2018, were included from a cohort of 2606 patients who had undergone laparoscopic gastric bypass surgery between January 1, 2011, and January 1, 2018. The CT scans were made for a suspicion of IH and structured reports were made using a standardised template with ten signs: (1) swirl sign, (2) small-bowel obstruction, (3) clustered loops, (4) mushroom sign, (5) hurricane eye sign, (6) small bowel behind the superior mesenteric artery, (7) right-sided anastomosis, (8) enlarged nodes, (9) venous congestion, and (10) mesenteric oedema. Furthermore, an overall impression of IH likelihood was given using a 5-point Likert scale. CT scans performed in 2011 until 2017, without structured reporting, were included for comparison. Sensitivity, specificity, positive predictive value (PPV), negative predictive value (NPV), and accuracy were calculated using two-way contingency tables; the chi-square test was used for calculating *p* value. Reoperation and 3-month follow-up were used as reference.

**Results:**

A total of 174 CT scans with structured reporting and 289 CT scans without structured reporting were included. Sensitivity was 81.3% (95% CI, 67.7–94.8%) and 79.5% (95% CI, 67.6–91.5%), respectively (*p* = 0.854); specificity was 95.8% (95% CI, 92.5–99.1%) and 88.6% (95% CI, 84.6–92.6%), respectively (*p* = 0.016); PPV was 81.3% (95% CI, 67.7–94.8%) and 55.6% (95% CI, 43.3–67.8%), respectively (*p* = 0.014); NPV was 95.8% (95% CI, 92.5–99.1%) and 96.0% (95% CI, 93.5–98.6%), respectively (*p* = 0.909); and accuracy was 93.1% (95% CI, 88.0–96.2%) and 87.2% (95% CI, 82.7–90.7%), respectively (*p* = 0.045).

**Conclusion:**

Structured reporting for the diagnosis of internal herniation after gastric bypass surgery improves accuracy and can be implemented in clinical practice with good results.

**Key Points:**

*• Ten signs are used to aid CT diagnosis of internal herniation after gastric bypass surgery.*

*• Structured reporting increases specificity and positive predictive value and thereby prevents unnecessary reoperations in patients without internal herniation.*

*• Structured reporting by means of a standardised template can help less experienced readers.*

## Introduction

With the worldwide increase of bariatric surgery, the occurrence of complications also increases. Internal herniation (IH) is one of these risks following gastric bypass surgery. An early and accurate diagnosis of IH is necessary to prevent potential ischaemic effects on bowel loops. CT scans are increasingly being used to enhance this diagnosis. To aid radiologists, different signs are described to differentiate between IH and normal postoperative anatomy [1–6]. However, these signs were never tested in clinical practice. In other diseases, structured reporting is found to help less experienced readers and improves surgeons’ confidence in the CT conclusion [7–9]. Due to increased completeness of reports, structured reporting improves oncological staging and surgical planning [10–13]. If structured reporting using CT signs improves accuracy of CT scans, unnecessary surgery might be prevented.

**Fig. 1:**
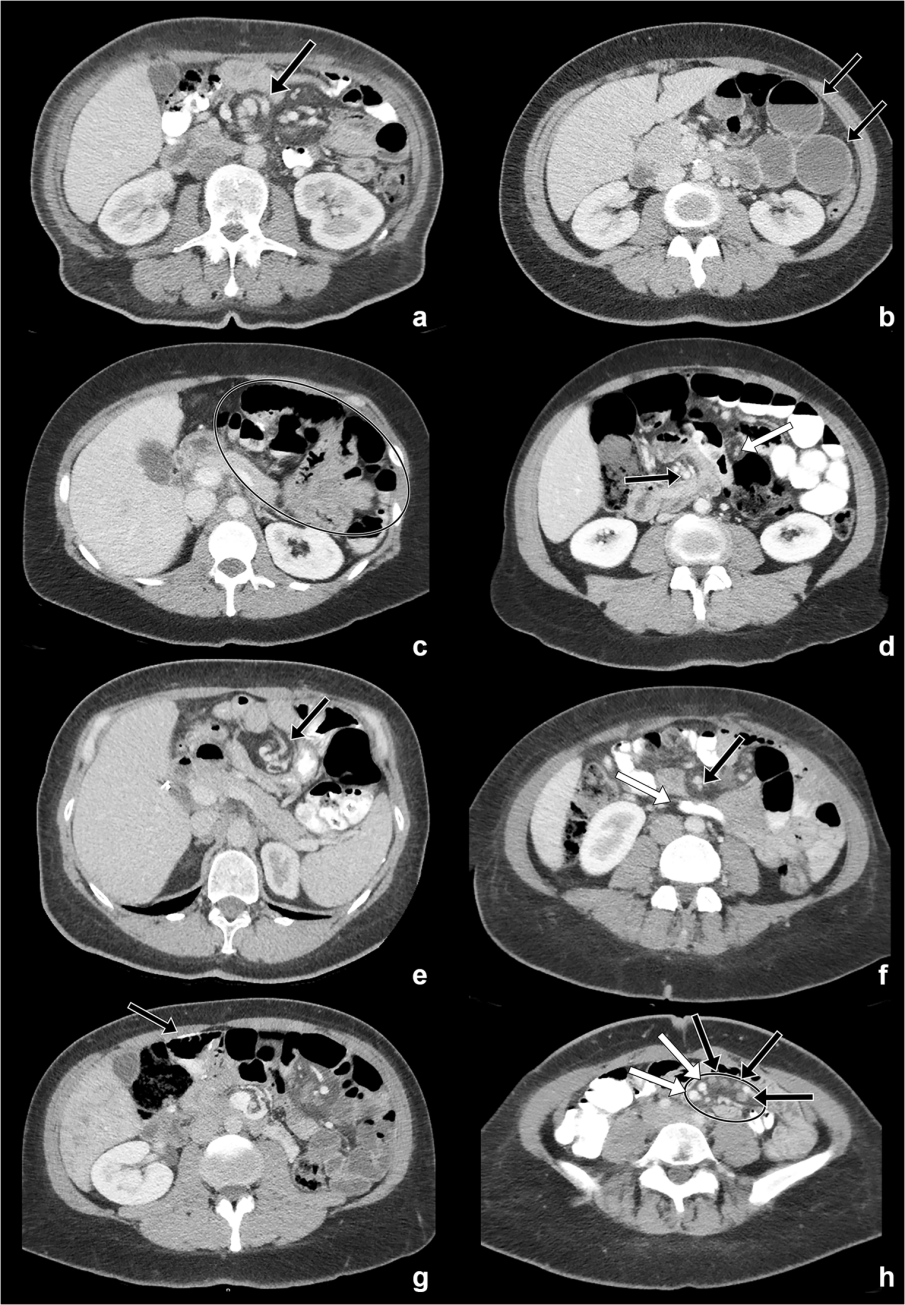
Enhanced axial CT images after intravenous and oral contrast through the midabdomen. **a** The swirl sign (arrow): rotation of the superior mesenteric artery and vein. **b** Small bowel obstruction (arrow): multiple dilated bowel loops with air-fluid levels. **c** Clustered bowel loops (circle): grouping of non-dilated small bowel near the abdominal wall. **d** Mushroom sign: protrusion of small bowel between the superior mesenteric artery (black arrow) and one of its branches (white arrow). **e** Hurricane eye sign (arrow): rotation of distal mesenteric fat and vessels closely surrounded by bowel. **f** Small bowel posterior to the superior mesenteric artery (SMA), other than the duodenum. Visible as a bowel (white arrow) behind the SMA (black arrow) caudal of the level of the duodenum, noticeable due to the low level in the abdomen with only a small part of the liver still visible. **g** A right-sided anastomosis (arrow): right-sided location of the distal jejunal anastomosis. **h** Enlarged lymph nodes (black arrows): multiple enlarged lymph nodes in the mesentery, venous congestion (white arrows): enlargement of the mesenteric veins compared with the corresponding arteries, and mesenteric oedema (circle): haziness of the mesenteric fat. Figure reprinted from Ederveen et al 2018 [14]

Recently, a study was performed into the accuracy of ten CT signs: (1) swirled appearance of mesenteric fat or vessels at the root of the mesentery (swirl sign), (2) dilated small-bowel loops with air-fluid levels as a sign of small-bowel obstruction, (3) clustered loops of small bowel, (4) mushroom shape of the herniated mesenteric root with protrusion of bowel between the superior mesenteric artery (SMA) and its branches (mushroom sign), (5) tubular or round shape of distal mesenteric fat closely surrounded by bowel loops (hurricane eye sign), (6) small bowel other than the duodenum passing posterior to the SMA, (7) right-sided location of the distal jejunal anastomosis, (8) the presence of enlarged mesenteric nodes as a secondary sign of lymphatic obstruction from mesenteric torsion (enlarged nodes), (9) tapering of mesenteric veins with subsequent engorgement (venous congestion, also referred to as beak sign), and (10) haziness of the mesenteric fat as a sign of mesenteric oedema (see Fig. 1) [14]. Structured reporting using these CT signs improved the specificity. Furthermore, no difference between experience levels was found. However, this study was performed on retrospectively obtained CT scans. To accurately study the effect of structured reporting, it should be implemented in clinical practice.

The aim of this study was to investigate if using ten CT signs in a structured reporting template in clinical practice improves accuracy of CT scans in case of a suspicion of IH, compared with non-structured free-text reporting previously performed.

## Materials and methods

This prospective study was performed in all patients who underwent laparoscopic gastric bypass surgery in our bariatric centre of excellence between January 1, 2011, and January 1, 2018. No patients were excluded. All consecutive CT scans for a suspicion of IH between June 1, 2017, and December 1, 2018, were prospectively included. Furthermore, all consecutive CT scans for a suspicion of IH between January 1, 2011, and January 1, 2017, were retrospectively included for comparison. All reoperation reports in patients undergoing a CT scan were screened to select reoperations for a suspicion of IH. Data on the presence of bowel herniation were extracted from surgery reports.

Episodes were considered IH positive if bowel herniation through a mesenteric defect was seen during reoperation within 90 days after the CT scan. An episode was considered negative when no IH was seen during surgery, or in case of negative clinical follow-up during a period of 90 days. The clinical follow-up was considered negative if no reoperation or no repeat CT scan was performed within these 90 days.

The local medical ethical review board approved the study design. Written informed consent was waived.

Part of the control group (1475 patients, undergoing gastric bypass surgery between 2011 and 2015) was previously reported in a study into the use of CT scans in case of suspicion of internal herniation and in a study into the accuracy of CT signs [14, 15].

### Surgical technique

All patients had undergone a laparoscopic Roux-en-Y gastric bypass (LRYGB) performed with an antecolic position of the jejunal limb. Both primary LRYGB and secondary LRYGB were included. Secondary LRYGB was performed on indication after sleeve gastrectomy, adjustable gastric banding, or vertical banded gastroplasty. Only from 2017 onwards, mesenteric and Petersen defects were closed routinely in our hospital.

### Image acquisition and analysis

All CT scans were performed with either a Brilliance iCT 256 slice or a Brilliance 64 CT scanner, with a slice thickness of 1 mm (Philips Medical). Multiplanar reconstructions were available. One litre of diluted oral contrast (Telebrix, Ioxithalamaat 6 mg I/mL; Guerbet) was administered 90 min before the CT scan if possible. All scans were performed 70 s after administration of 100 mL intravenous contrast Iomeron (Iomeprol 300 mg I/mL; Bracco Imaging).

All CT scans between June 1, 2017, and December 1, 2018, were rated in clinical practice by the attending radiologist or in-training radiologist (under the supervision of a radiologist) using a standardised template stating ten signs. They had access to the clinical information provided by the clinicians. The ten signs used were (1) swirl sign, (2) small-bowel obstruction, (3) clustered loops of small bowel, (4) mushroom sign, (5) hurricane eye sign, (6) small bowel posterior to the SMA, (7) right-sided distal anastomosis, (8) enlarged nodes, (9) venous congestion, and (10) mesenteric oedema. Each sign was scored as being present or absent. Furthermore, an overall impression was given using a 5-point Likert scale, 1 being definitely no IH, 5 being definite IH.

If the standardised structured reporting template was not used in the study period June 2017 until December 2018, the CT scans were excluded from further analysis.

For the control group, the radiologic report and conclusion of the included CT scans were analysed for the use of CT signs and the overall impression. The reports were made by a radiologist or an in-training radiologist (under the supervision of a radiologist) without structured use of the CT signs. They had access to the clinical information provided by the clinicians.

CT scans performed in other hospitals and revised in the study institution were included in this study as well.

### Statistical analysis

All data were entered into a computerised spreadsheet (Excel; Microsoft) and were analysed with Statistical Package for Social Sciences 21.0 (SPSS Inc.). Patient characteristics are listed as mean (± SD) or median (interquartile range (IQR) (25th–75th percentile)), depending on the normality of the distribution. Continuous data were compared with the independent sample *t* test or Mann-Whitney test for not normally distributed data. Categorical data were compared with the chi-square test or Fisher’s exact test. The significance level was set at *p* < 0.05. The sensitivity, specificity, positive predictive value (PPV), negative predictive value (NPV), and accuracy for overall impression were calculated using two-way contingency tables. A 5-point Likert scale was used to score overall impression. The scale was digitised, considering 1–3, negative for IH and 4–5, positive.

## Results

### Demographics

A total of 2606 patients underwent gastric bypass surgery in the study period, 83.7% were female with a mean age of 43.3 (± 10.5) years and a median body mass index (BMI) of 41.5 kg/m^2^ (IQR 38.9–44.7 kg/m^2^) (see Table 1). In the study population, a total of 477 CT scans were performed in 354 patients. Patient characteristics are summarised in Table 1. The median follow-up time after CT scan was 31 months (IQR 13–52 months). The median time interval from gastric bypass until CT scan with an enquiry of IH was 555 days (IQR 276–1008 days).

**Table 1:** Patient demographics. *SD* standard deviation, *BMI* body mass index, *IQR* interquartile range (25th–75th percentile), *AGB* adjustable gastric banding, *VBG* vertical banded gastroplasty

Characteristic	Total population (*n* = 2606)	Patients with CT scan (*n* = 354)	Patients without CT scan (*n* = 2252)	*p* value
Sex				0.224^3^
Male, *n* (%)	426 (16.3)	50 (14.1)	376 (16.7)	
Female, *n* (%)	2180 (83.7)	304 (85.9)	1876 (83.3)	
Age, year, mean (± SD)	43.3 (10.5)	40.7 (10.5)	43.7 (10.5)	< 0.001^4^
Initial BMI, kg/m^2^, median (IQR)	41.5 (38.9–44.7)^1^	41.1 (38.4–45.0)	41.6 (39.0–44.6)^2^	0.188^5^
Operation				< 0.001^3^
Primary, *n* (%)	1918 (73.6)	243 (68.6)	1675 (74.4)	
Secondary after sleeve, *n* (%)	254 (9.7)	62 (17.5)	192 (8.5)	
Secondary after AGB, *n* (%)	308 (11.8)	35 (9.9)	273 (12.1)	
Secondary after VBG, *n* (%)	126 (4.8)	14 (4.0)	112 (5.0)	

^1^2556 patients

^2^2202 patients

^3^*p* value calculated using the chi-square test

^4^*p* value calculated using the independent samples *t* test

^5^*p* value calculated using the Mann-Whitney *U* test

### CT scans with structured reporting

**Fig. 2:**
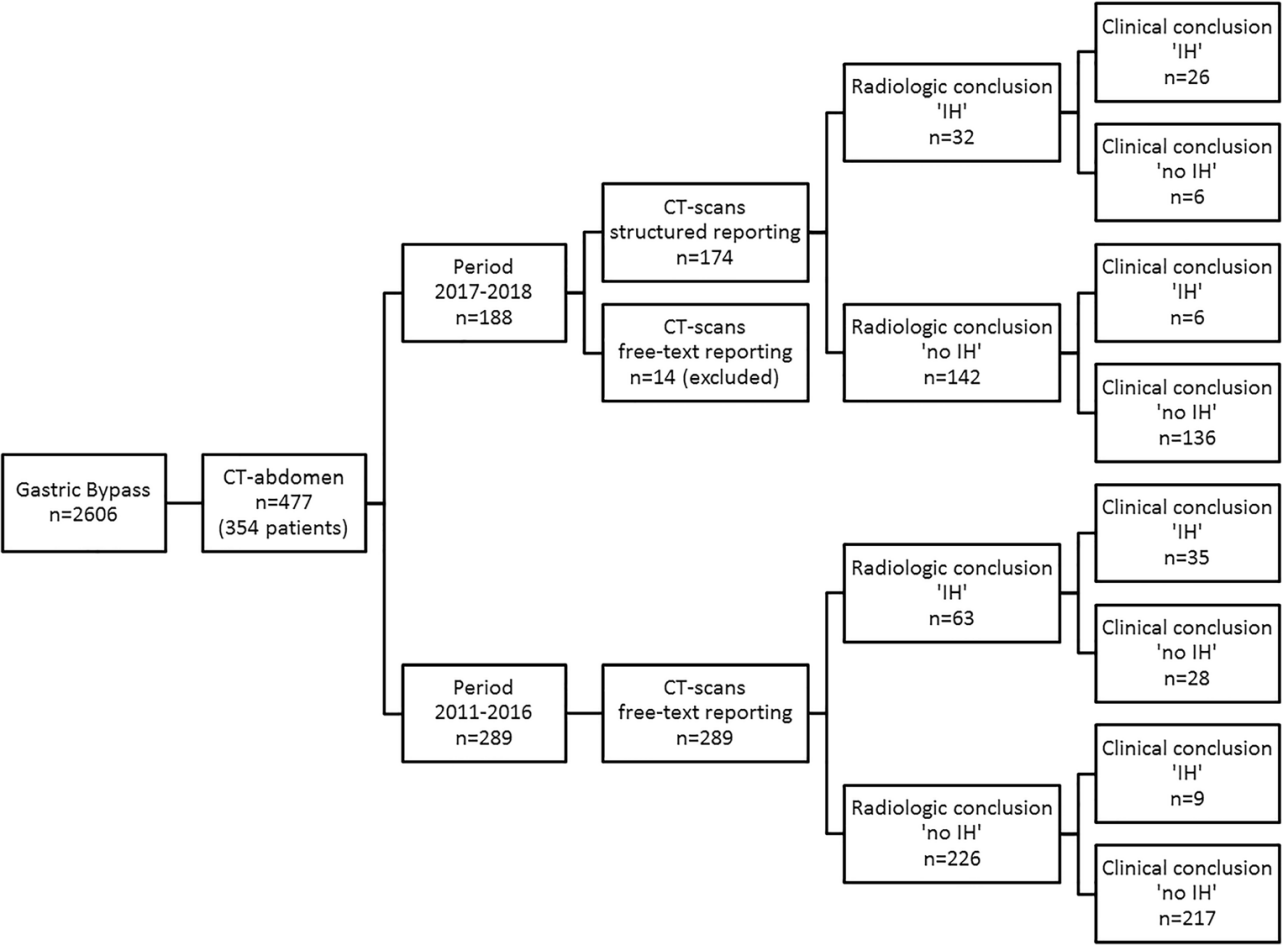
Flowchart of patient and CT scan inclusion in the study and outcomes of episodes. Clinical conclusion is the final diagnosis at the surgical intervention or after 90 days of follow-up

In the time period June 1, 2017, until December 1, 2018, 188 CT scans performed in 166 patients were prospectively included in this study (see Fig. 2). The structured reporting template was used in 92.6% (174/188). The other 14 CT scans were excluded from further analysis. After the CT scans with structured reporting, 58 reoperations were performed, with a median of 1 day after CT scan (IQR 0–27 days). In 53.4% (31/58) of all these operations, an IH was confirmed.

### CT scans with free-text reporting

A total of 289 CT scans in 226 patients were included in the time period January 1, 2011, and January 1, 2017 (see Fig. 2). After these CT scans, 80 reoperations were performed, with a median of 5 days after CT scan (IQR 1–24 days). In 55.0% (44/80) of all these operations, an IH was confirmed.

### Comparison between free-text and structured reporting

Using the conclusion of structured reporting resulted in a sensitivity and specificity for IH of 81.3% (26/32) and 95.8% (136/142), respectively. Using the conclusion of the free-text reporting resulted in a sensitivity and specificity for IH of 79.5% (35/44) and 88.6% (217/245), respectively. Improvement of specificity was significant (*p* = 0.016). Also the accuracy improved from 87.2% (252/289) in free-text reporting to 93.1% (162/174) in structured reporting (*p* = 0.045) (see Table 2).

**Table 2:** Comparison of structured reporting vs. free-text reporting. Values are numerator/denominator (%; 95% confidence interval). *P* value was calculated using the chi-square test

	Structured reporting	Free-text reporting	*p* value
Sensitivity	26/32 (81.3; 67.7–94.8)	35/44 (79.5; 67.6–91.5)	0.854
Specificity	136/142 (95.8; 92.5–99.1)	217/245 (88.6; 84.6–92.6)	0.016
Positive predictive value	26/32 (81.3; 67.7–94.8)	35/63 (55.6; 43.3–67.8)	0.014
Negative predictive value	136/142 (95.8; 92.5–99.1)	217/226 (96.0; 93.5–98.6)	0.909
Accuracy	162/174 (93.1; 88.0–96.2)	252/289 (87.2; 82.7–90.7)	0.045

Table 3 shows the accuracy of the different signs when structured reporting was used. Table 4 shows the overall impression in terms of Likert scale.

**Table 3:** Signs and overall impression in structured reporting. Values are numerator/denominator (%; 95% confidence interval). *SMA* superior mesenteric artery

Sign	Sensitivity	Specificity	Accuracy
Swirl sign	21/32 (65.6; 49.2–82.1)	135/142 (95.1; 91.5–98.6)	156/174 (89.7; 83.9–93.6)
Small bowel obstruction	5/32 (15.6; 3.0–28.2)	134/142 (94.4; 90.6–98.2)	139/174 (79.9; 73.0–85.4)
Clustered loops	8/32 (25.0; 10.0–40.0)	128/142 (90.1; 85.2–95.0)	136/174 (78.2; 71.1–83.9)
Mushroom sign	11/32 (34.4; 17.9–50.8)	140/142 (98.6; 96.7–100)	151/174 (86.8; 80.6–91.3)
Hurricane sign	12/32 (37.5; 20.7–54.3)	141/142 (99.3; 97.9–100)	153/174 (87.9; 81.9–92.2)
Small bowel behind the SMA	7/32 (21.9; 7.6–36.2)	139/142 (97.9; 95.5–100)	146/174 (83.9; 77.4–88.9)
Right-sided anastomosis	1/32 (3.1; 0–9.2)	141/142 (99.3; 97.9–100)	142/174 (81.6; 74.9–86.9)
Enlarged nodes	17/32 (53.1; 35.8–70.4)	119/142 (83.8; 77.7–89.9)	136/174 (78.2; 71.1–83.9)
Venous congestion	26/32 (81.3; 67.7–94.8)	134/142 (94.4; 90.6–98.2)	160/174 (92.0; 86.6–95.4)
Mesenteric oedema	22/32 (68.8; 52.7–84.8)	128/142 (90.1; 85.2–95.0)	150/174 (86.2; 80.0–90.8)

**Table 4:** Positive predictive value of the Likert scale. *IH* internal herniation, *PPV* positive predictive value, *CI* confidence interval

Overall impression	IH (*n*)	No IH (*n*)	PPV (%, (95% CI))
1	3	113	2.6 (0.7–7.9)
2	2	17	10.5 (1.9–34.5)
3	2	6	25.0 (0.4–64.4)
4	14	4	77.8 (51.9–92.6)
5	11	2	84.6 (53.7–97.3)

During structured reporting, all signs were used per definition in 100% of the reports with the standardised template. Table 5 shows the signs mentioned in free-text reporting.

**Table 5:** Signs mentioned in free-text reporting. *SMA* superior mesenteric artery

Sign	Mentioned	Not mentioned	Percentage
Swirl sign	170	119	58.8
Small bowel obstruction	156	133	54.0
Clustered loops	53	236	18.3
Mushroom sign	4	285	1.4
Hurricane sign	2	287	0.7
Small bowel behind SMA	2	287	0.7
Right-sided anastomosis	27	262	9.3
Enlarged nodes	94	195	32.5
Venous congestion	32	257	11.1
Mesenteric oedema	93	196	32.2

## Discussion

The aim of this study was to investigate if using structured reporting with the use of ten CT signs in clinical practice improves accuracy of CT scans in case of a suspicion of IH, compared with free-text reporting. This study found that the use of ten CT signs improves accuracy in diagnosing IH on CT scans from 87.2 to 93.1% (*p* = 0.045). Furthermore, an increase in specificity and positive predictive value was found from 88.6 to 95.8% (*p* = 0.016) and from 55.6 to 81.3% (*p* = 0.014), respectively, hereby reducing the number of unnecessary laparoscopies.

Our findings confirm the assumptions of previous studies, that also found that structured reporting using CT signs improved accuracy [14, 16]. However, these previous studies performed blind reading of the CT scans retrospectively and compared them with the original report. To our knowledge, this is the first study to prospectively use structured reporting in clinical practice for the diagnosis of IH.

The accuracy of the different signs can only be compared to retrospectively performed studies [1–5, 14, 16]. The range of sensitivity and specificity in and between these studies is broad. Most noteworthy, the sensitivity of the swirl sign decreased compared to most previous studies with a comparable increased specificity. However, the accuracy is comparable to a previous study into the ten CT signs, 89.7% compared to 86.5–91.4% [14]. The accuracy of small bowel obstruction, clustered loops, right-sided anastomosis, and enlarged nodes decreased compared to this previous retrospective study. Of these signs, the sensitivity is rather poor and also the accuracy is not excellent. The decrease in accuracy might be explained due to the variety of (in-training) radiologists who reported the CT scans in the prospective setting. The poor sensitivity of these signs suggests these might not be valuable in clinical practice and could be excluded from the structured reporting. In the retrospective setting of this study, most of these signs were also infrequently mentioned.

The use of Likert scale in determining the possibility of IH gives a positive predictive value of 25.0% for the “doubt category” of 3. This means that radiologic doubt is in most cases not an IH. An independent experienced radiologist (SJ) blindly reviewed the nine CT scans with a Likert scale of 3. She diagnosed one IH correctly; however, she missed the other IH. Furthermore, one CT scan remained in category 3. All other CT scans previously given a 3 were found to be low suspect for IH. Having an experienced radiologist as second opinion in case of doubt gives more certainty and thereby might prevent unnecessary surgeries.

In the current study, the ten most described signs in recent literature were used for the structured reporting template [1–6]. Some signs previously described differently in various articles were combined to one sign in our template. We found that the mushroom and hurricane eye signs were minimally sensitive, however had good specificity, PPV, and NPV. Other minimally sensitive signs as clustered loops, small bowel behind the SMA, right-sided anastomosis, and enlarged nodes had also poorer PPV and NPV. Future research into the usefulness of all CT signs might be performed. Maybe not all currently used signs are necessary, or maybe other signs should be included in the structured reporting template.

Our study has several strengths and limitations. This study is the first prospective study into the use of CT signs for diagnosing IH in clinical practice.

A limitation of this study is that radiologists in our hospital might be more aware of the signs that can be used for diagnosing IH on CT scans because of previous research on this subject performed in our hospital. To minimise this effect, we excluded the time period January 2017 until June 2017. In May 2017, the signs were explained to all (in-training) radiologists and the implementation of the structured reporting was started. However, bias due to improvement in technical skills of the radiologists cannot be completely excluded. This bias is reduced because also new (in-training) radiologists, with minimal knowledge of IH and the signs, started working with the structured reporting template. Moreover, the in-training radiologists were the main reporters of the CT scans with suspicion of IH. Another drawback is the fact that structured reporting was not used on all CT scans. This was mostly caused by an initial lack of knowledge of the structured reporting template in newly hired (in-training) radiologists. Surgeons in our hospital were also more aware of the diagnostic support radiologists could provide and more frequently requested CT scans before considering a reoperation. Due to the clear radiological reports with the use of standardised format, they might also be more inclined to rely on the radiological diagnosis, changing the reoperation considerations.

To increase the amount of includable patients in the accuracy analysis, 90 days of follow-up was added as reference for the CT scan diagnosis. The cut-off of 90 days was chosen because an IH was considered unlikely after 90 days without further intervention. This cut-off of 90 days is debatable, but in this study, it was believed to be reasonable.

To conclude, this study found an increase in accuracy when using structured reporting in clinical practice compared with free-text reporting in diagnosing internal herniation on CT scans after gastric bypass surgery. Also, the found increase in positive predictive value can prevent unnecessary reoperations in patients with low probability of IH. The use of structured reporting can possibly improve certainty of diagnosing IH on CT scans in less-experienced (in-training) radiologists.

## Abbreviations

AGB: Adjustable gastric banding
BMI: Body mass index
IH: Internal herniation
IQR: Interquartile range
LRYGB: Laparoscopic Roux-en-Y gastric bypass
NPV: Negative predictive value
PPV: Positive predictive value
SD: Standard deviation
SMA: Superior mesenteric artery
VBG: Vertical banded gastroplasty
